# Assessment of Vegetable Species for Microgreen Production in Unheated Greenhouses: Yield, Nutritional Composition, and Sensory Perception

**DOI:** 10.3390/plants13192787

**Published:** 2024-10-04

**Authors:** Pabla Rebolledo, Gilda Carrasco, Claudia Moggia, Pedro Gajardo, Gabriela Rodrigues Sant’Ana, Fernando Fuentes-Peñailillo, Miguel Urrestarazu, Eduardo Pradi Vendruscolo

**Affiliations:** 1 Departamento de Horticultura, Facultad de Ciencias Agrarias, Universidad de Talca, Talca 3460000, Chile; prebolle@utalca.cl (P.R.); cmoggia@utalca.cl (C.M.); pgajardo19@alumnos.utalca.cl (P.G.); 2 Departamento de Agronomia, Universidade Estadual de Mato Grosso do Sul, Cassilândia 79540-000, Brazil; gabrielasant_ana@icloud.com; 3 Instituto de Investigación Interdisciplinaria (I3), Vicerrectoría Académica (VRA), Universidad de Talca, Talca 3460000, Chile; ffuentesp@utalca.cl; 4 Departamento Agronomía, Universidad de Almería, 04120 Almería, Spain; mgavilan@ual.es

**Keywords:** microgreens, vegetables, food composition, sensory analysis, functional foods

## Abstract

Cultivating microgreens in central-southern Chile in unheated greenhouses offers a viable and productive alternative to growers. In 2023, two experiments were conducted in autumn and spring. These experiments involved the production of microgreens of eleven vegetable species. The tray system with the substrate was employed. Subsequently, agronomic, nutritional, and sensory perception variables were assessed. Despite notable fluctuations in external temperatures between these seasons, a diverse array of microgreens can be successfully cultivated, meeting local consumer preferences. Research indicates that microgreens grown under these conditions exhibit high nutritional quality, serving as a rich source of essential nutrients and bioactive compounds beneficial to human health. This nutritional value remains consistent across autumn and spring, establishing microgreens as a reliable and valuable food option. The observed acceptance and purchasing intentions among the surveyed population suggest a promising market opportunity for introducing these products regionally. Consumers appreciate microgreens’ quality and nutritional advantages, underscoring their potential.

## 1. Introduction

Microgreens, also known as microplants, are young vegetables or herbs characterized by fully expanded cotyledon leaves and the emergence or partial expansion of the first true leaf [1,2]. They originated in California, United States, in the 1980s and were initially grown commercially. The surge in marketing efforts was due to their remarkable taste, colors, appearances, and textures [3]. They can be used in salads, soups, sandwiches, or as visually appealing, edible garnishes, enhancing various dishes [4]. Additionally, microgreens are rich in diverse nutrients, driving increased consumer interest due to their nutritional value [1,5].

Microgreens are rich in minerals and phytochemicals that promote human health, containing higher nutrient levels than adult plants and earning them the label of superfoods or functional foods. Numerous studies have underscored their vital impact on human health [1,6,7,8]. In Chile, the cultivation of microgreens has markedly increased in recent years, especially in urban areas with a rising demand for fresh, nutritious products [3]. Recent reviews, such as [9], have highlighted the nutritional value of various microgreen species, emphasizing that their nutrient composition and content can significantly vary based on the growth stage and are influenced by factors such as cultivars and growing conditions [10,11]. Additionally, microgreens possess superior phytochemical profiles compared to baby vegetables, making them an even more potent source of health-promoting compounds [12].

Microgreen production is advantageous for productivity as it optimizes physical space and inputs, resulting in minimal resource losses [13]. This demonstrates the increased sustainability of microgreen production compared to the traditional cultivation of mature plants [14,15]. Moreover, microgreen production allows for a diverse range of species to be cultivated and quickly commercialized due to their short shelf-life. Consequently, the cultivation environment must be close to consumer centers [16]. As various methodologies and technologies continue to gain prominence, there is a growing need to enhance the approaches used in producing microgreens. Essentially, evaluating the cultivation of different species under accessible conditions encourages local investments, bolsters the food supply, and elevates quality. Furthermore, conducting assessments on mineral content and consumer preferences leads to better reception in the local market, enhancing commercialization and helping producers to direct their activities.

This study aimed to evaluate the performance and nutritional composition of various microgreens produced locally and assess consumer perception regarding their organoleptic characteristics.

## 2. Results

### 2.1. Earliness and Yield

During the study, only chard had a low germination rate (20%), while the other species had germination rates above 80%. Additionally, the sowing density ranged from 90 to 640 mg m^−2^, and the average germination time varied between three and nine days due to the distinct characteristics of each species (Table 1).

### 2.2. Biometric and Productive Analysis

In the autumn experiment, radishes had significantly greater height than other species, while carrots and lettuce had the lowest average for plant height (Table 2). In the spring experiment, mizuna, fennel, and broccoli showed no significant differences in plant height, and all three species were taller than spinach. However, anise exhibited growth issues, which were attributable to the elevated temperatures recorded within the greenhouse during the spring period. Additionally, regarding green mass harvested per tray and productivity, radish and lettuce exhibited superiority in the autumn experiment, whereas mizuna and broccoli excelled in the spring experiment (Table 2).

### 2.3. Nutritional and Mineralogical Analysis

It was found that the total proteins and raw fiber contents of the species used in the autumn experiment varied between them, ranging from 3.7% to 4.8% and 0.4% to 0.7%, respectively. For these same characteristics, the variation presented in the spring experiment was 2.42% to 3.50% and 0.5% to 0.6%, respectively, with higher averages of proteins and fiber being observed for fennel (Table 3).

For total phenol contents, two groups were observed: one formed by chard, carrot, kale, and arugula, with values above 3000 mg kg^−1^ GAE, which was higher than the second group, formed by radish and lettuce, for which values close to 2000 mg kg^−1^ GAE were observed. In the spring experiment, the highest value of total phenol content was observed for spinach (11,000 mg kg^−1^), followed by fennel, mizuna, and broccoli (4500 to 6000 mg kg^−1^), respectively.

Only radish, with the lowest average, showed a significant difference from the other species for the nitrate characteristic in the autumn. Chard stood out for beta-carotene levels, with values over twice the concentrations verified for nitrate.

In the autumn experiment, only radish showed significantly lower average beta-carotene levels than other species. On the other hand, chard stood out with beta-carotene levels over twice the concentrations found in the other species. In the spring experiment, fennel and spinach had the highest average nitrate and beta-carotene levels, respectively. These characteristics varied across species, ranging from 1733.33 mg L^−1^ to 2178 mg g^−1^ (Table 3).

Except for P content, significant differences existed in the mineralogical compositions of the species used in the autumn experiment. In this context, kale microgreens contained higher Fe, Mg, and Ca levels, while chard showed higher Na levels, surpassing only radish (Table 4). Among the species in the spring experiment, mizuna microgreens exhibited the highest Fe, Mg, and Ca concentrations, while spinach had the highest average Na content. The variations in Fe, Mg, Ca, Na, and P content in the spring experiment were 45.46 mg kg^−1^, 84.3 mg kg^−1^, 594.2 mg kg^−1^, and 25.55 mg kg^−1^, respectively, and the number of sample repetitions did not allow for a comparison of means. (Table 4).

### 2.4. Organoleptic Properties and Preference for Purchasing Vegetables

Significant differences were observed in the various species’ appearance in the autumn and spring experiments. In the autumn experiment, chard was rated as having an extremely pleasant appearance, while carrot and arugula received the worst evaluations. Similarly, mizuna had the best appearance in the spring experiment, while spinach had the worst. Despite these differences, none of the species received scores equal to or greater than 5, which could indicate negative evaluations.

In both experiments, color differences were observed between species. According to the respondents, chard and Mizuna consistently had the best color perception.

For texture, there were no differences between species in the autumn experiment. However, significant differences were observed in the spring experiment, with spinach having the most pleasant texture and receiving the highest evaluation (Table 5).

Notably, for the appearance, color, and texture characteristics, all averages obtained resulted in a positive evaluation of the species since, according to the scale used, the evaluations remained very pleasant and moderately pleasant (values vary from 2.2 to 3.2). However, for the flavor characteristic, the evaluators’ scores varied between moderately pleasant and slightly pleasant (values range from 3.2 to 4.5 in the autumn experiment), with radish and arugula the species that received the worst evaluations, while kale was considered the tastiest. For the spring experiment, spinach and fennel differed significantly, with spinach being considered the species with the best flavor (Table 5).

Regarding purchase intention, among the species used in the autumn experiment, there was no difference between the average scores of the evaluators. Thus, the variation between 2.18 and 2.84 implied a purchase intention result of “would probably buy” (Table 6). In addition, for the spring experiment, it was found that mizuna had a significantly lower average than fennel and spinach, receiving a purchase intention of “I would definitely buy it”, while the other species had the same purchase intention as the species in the experiment autumn, “I would probably buy” (Table 6).

In the autumn experiment, of the total number of respondents, 53% were men and 47% were women, with most aged between 20 and 25 (79%). Additionally, 65% of the judges declared that they frequently consumed vegetables, but 84% responded that they had never consumed microgreens. In the spring experiment, of the total number of respondents, 68% were men and 32% were women, the majority of whom were between 20 and 25 years old (68%); from the total, 68% were frequent vegetable consumers, but 55% had never consumed microgreens.

### 2.5. Correlation Analysis

The search for direct relationships between the quality of microgreens and consumers’ acceptance of them can help define production planning. In this way, a correlation analysis was carried out to determine which characteristics could positively interfere with the choice of a certain species. It is also possible to determine which characteristics do not affect sensory evaluations or which could negatively impact the choice. While red represents significant interactions, these are related to sensory evaluations, with lower averages indicating positive evaluations. In this sense, all the interactions highlighted with thick lines seem positive, with interference between the factors of Na, Fe, beta-carotene content, and the appearance of the species.

**Figure 1:**
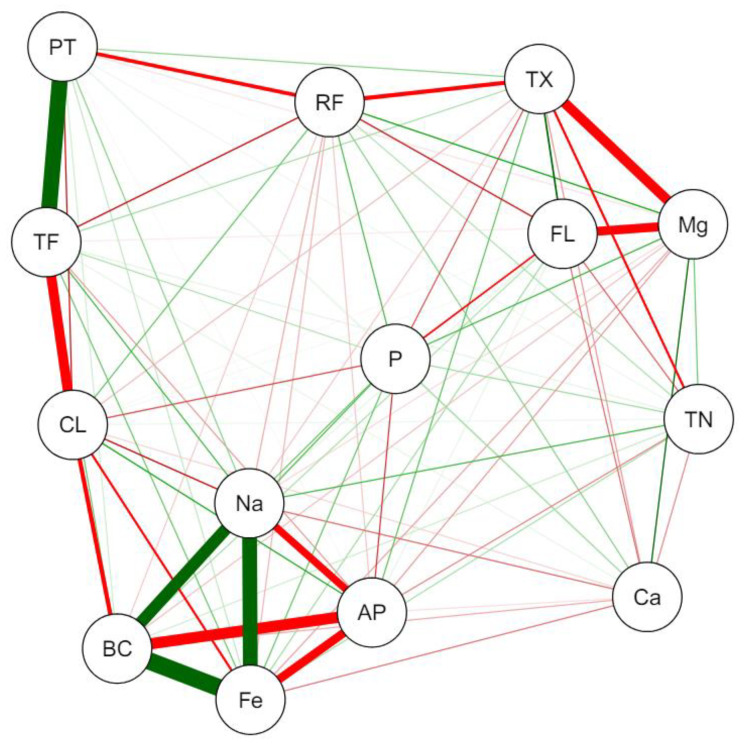
Pearson correlation network between the variables of total protein (TP), total phenols (TF), nitrates (TN), beta-carotene (BC), raw fiber (RF), iron (Fe), manganese (Mg), calcium (Ca), sodium (Na), phosphorus (P), appearance (AP), color (CL), texture (TX), and flavor (FL) of different species of microgreens evaluated during the autumn experiment. Positive correlations were highlighted in green, while negative correlations were highlighted in red; determining the line thickness followed a cut-off value of 0.7, corresponding to 70% reliability.

Mg levels interfere with texture and flavor characteristics, and there is a direct relationship between total proteins and total phenols, the latter with the plants’ characteristic color (Figure 1). For all the correlation groups formed, there is evidence that the variation in the composition of species, nutrients, and bromatological components significantly alters the sensory evaluations of the individuals participating in the autumn experiment.

## 3. Discussion

### 3.1. Biometric and Productive Analysis

Notably, there is great variation in development among the different species used to grow microgreens (Table 2). This fact, combined with the environmental conditions of cultivation, will define a set of interactions encompassing sowing and harvesting cycles. In this sense, using unheated greenhouses, common environments for vegetable producers, creates a reliance on local environmental conditions, leading to increased variability in temperature and relative air humidity.

The variability in height development between species is related to intrinsic genetic and morpho-physiological factors. Species have different characteristics regarding the amount of seed reserves: for example, lettuce, has small seeds and low reserves, compared to radish, which has larger seeds, enabling the development of larger seedlings. However, the values obtained are based on height values previously reported in the literature for species cultivated in different environmental conditions [17,18,19].

During the autumn experiment, low temperatures may explain the lengthening of the production cycle, 33 days. The cultivation period was characterized by average daily temperatures below 20 °C, with around 50% of days with average temperatures below 15 °C. In this sense, harvests are reported to be carried out between 4 and 12 days after sowing for some of the species used in the present study, such as radish, chard, and spinach [20]. However, such studies are mostly carried out in controlled environmental chambers, optimizing the conditions for plant development. In addition to temperature, other environmental factors can also significantly interfere with cultivation, such as incident radiation, influencing crop productivity averages, as seen in the present study (Table 2), in which the average production obtained in autumn cultivation was 5.7 times lower than that obtained in spring. Such characteristics greatly influence the development of crops, resulting in seasonality for species such as chard [21] and arugula [10], for which it appears that autumn crops, when the temperature and incident radiation are decreasing, are less productive than crops in spring when temperature and radiation are rising.

### 3.2. Nutritional and Mineralogical Analysis

Plants harvested early, especially microgreens, are considered bioactive foods or superfoods due to their high levels of elements beneficial to human health [10]. In this sense, other studies have demonstrated that nutritional elements and bioactive compounds are found in higher microgreens values than conventionally harvested vegetables [11,22].

The protein levels in this research did not show differences between the species evaluated in the autumn experiment, ranging between 3.66% and 4.75%. In the spring experiment, the values ranged between 2.4% and 2.9%. The literature reports protein values like those obtained in this study, and in some cases, they exceed the ones previously reported (Table 3), as with radish, carrot [23], and arugula [24]. No variations were observed in this study’s protein, phenols, and fiber contents, and the variation in the nitrate contents was confirmed (Table 3).

Among the species, significant differences were only found in the autumn experiment, with the lowest nitrate content recorded for radish (1099.55 mg L^−1^) and the highest concentration observed for lettuce (4169.27 mg L^−1^). For the spring experiment, the lowest value was found for spinach (3866.67 mg L^−1^), while the highest concentration was verified for fennel (5600.00 mg L^−1^). High variability in nitrate levels is close to those previously verified by other authors, in which different species vary between 13 and 8279 mg kg^−1^, which are also influenced by the growing season [25]. High variability in nitrate levels can also occur in species belonging to the same botanical family, as verified in the present study (Table 3), which may be related to genetic factors and cultivation environment and inputs used, which are also influenced by the growing season [25]. In addition, due to the negative implications of consuming high nitrate levels, it is essential to find alternatives to reduce these levels in microgreens.

Beta-carotene is a red-orange pigment found in fruits and vegetables; it is a precursor of vitamin A and has an important physiological role in humans, such as vision and development. Also, it works as an important antioxidant that allows the maintenance of cellular metabolism [5]. This compound can be obtained abundantly in microgreens, implying that consuming these young plants can help prevent disorders and diseases, increasing the quality of life [9]. Therefore, an increase in beta-carotene levels is desired in microgreens. Significant variation was also observed for this compound between the species studied, with the highest concentrations of chard, fennel, and spinach (Table 4). These three species have practically double the concentration of beta-carotene compared to the other species evaluated. Just like beta-carotene, the presence of minerals in species used for producing microgreens can benefit human health, allowing the natural replacement of minerals and avoiding problems related to their absence [9,25]. These characteristics make microgreens foods that promote human health and which, in the future, can be included in specific diets to reduce problems related to inflammatory and cardiovascular diseases and obesity, among others. The variation observed between species in terms of their mineral composition (Table 4) implies that different species, or even genetic materials from the same species, can be used in a specific way for a given need. The literature shows a positive relationship between early harvesting and the amount of nutrients available, with microgreens tending to contain higher amounts of minerals [26]. Also, combining species could result in an even richer food, generally increasing the nutrients available to the final consumer.

### 3.3. Organoleptic Properties and Preference for Purchasing Vegetables

Sensory perception is something particular and varies between people according to their perception of what is pleasant or unpleasant. In this sense, the sensory evaluation of the species of microgreens used in this study, in both experiments, was considered positive since there were no average measurements of scores above 5 points (Table 5). Thus, it would be verified if evaluators were indifferent to characteristics and arugula, which obtained the highest averages in the flavor assessment, followed by fennel, belonging to the Apiaceae family, which has a pronounced aromatic characteristic [27].

**Figure 2:**
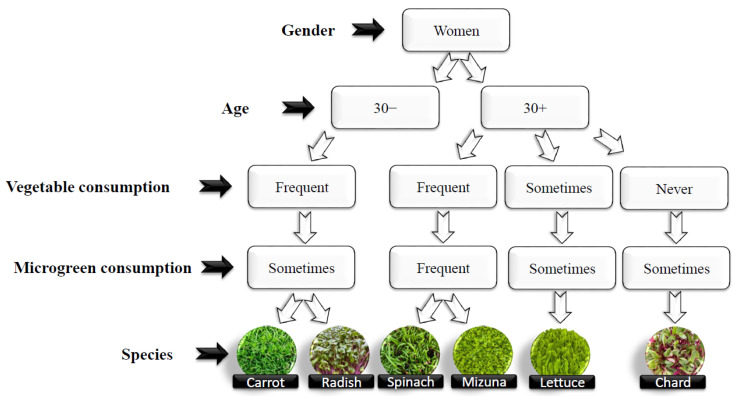
Map of female reviewers’ purchase intention.

Bitterness can also trigger an aversion reaction to the product due to evolutionary issues since it can be related to toxic compounds in nature [6]. The highest averages were verified for the flavor characteristic. This is related to the fact that some species used in producing microgreens may present marked spiciness or bitterness due to the high levels of compounds present, such as sulfur in the case of some species. This positive evaluation also resulted in positive purchase intention for all species evaluated, varying between 1.95 for mizuna and 2.84 for carrots on the 1 to 9 scale used. It was also found that purchase intention evaluations formed different groups; thus, the species that received more positive evaluations were mainly women over 30 years old. Moreover, those who claimed to consume vegetables and microgreens frequently or sometimes also evaluated two (spinach and mizuna) of the eight species used more positively (Figure 2). In this sense, it is also observed that groups of younger women who frequently consume vegetables and microgreens tend to opt for species with a more robust flavor (Table 5).

**Figure 3:**
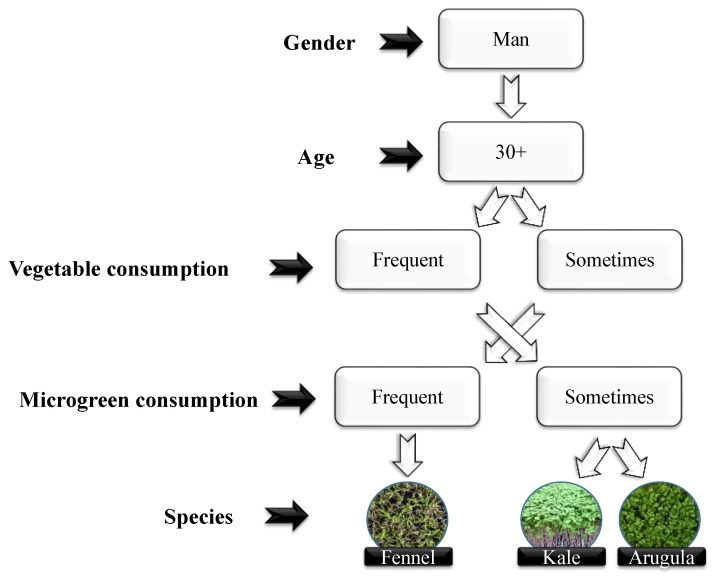
Map of male reviewers’ purchase intention.

Similar to those observed for women, the evaluations of the group of men also indicated that the best evaluations were concentrated on evaluators over 30 years old and who habitually consume vegetables regularly (Figure 3). For this group, we also found that two of the three species indicated as the ones with main purchase intentions, fennel and arugula, received high evaluations regarding flavor (Table 5), indicating a preference for these evaluators over species with a strong flavor.

### 3.4. Correlation Analysis

Our findings enabled us to identify relationships between microgreens’ composition and evaluators’ sensory perception in the autumn experiment (Figure 1). In this sense, we can highlight that the presence of higher levels of beta-carotene results in better evaluations of the general appearance of microgreens. This fact may be related to the color of the microgreens, as more intense colors tend to influence consumers’ decision-making [28].

For other species consumed as mature vegetables, it appears that the presence of beta carotene increases consumer acceptability, increasing evaluations regarding flavor and general sensory characteristics, such as tomatoes [29]. Consumers also gave higher scores in sensory evaluation to orange sweet potatoes, rich in beta carotene, when compared to those with purple and yellow colors [30], a result like that observed for cassava cultivars, in which biofortification with beta-carotene positively influenced greater acceptability on the part of the evaluators, who also related part of their choices to the more intense orange color [31]. This relationship between beta-carotene content and consumer acceptability can have important implications regarding the definitions of species to be cultivated, increasing commercialization potential. In addition to economic factors, using species-rich compounds in this compound can benefit consumers, improving the general characteristics of food quality [5]. Also, the positive relationships between magnesium texture and magnesium flavor may imply the choice of vegetables rich in this nutrient, which participates in a series of metabolic processes in the human body, improving performance, balancing blood sugar levels, and promoting heart health, among others [32].

In sensory terms, as demonstrated in this research (Figure 1), it can be seen in the literature that magnesium is related to the flavor and texture of a series of vegetables, as it is involved in metabolic activities that will influence the levels of sugars in vegetables, as well as on texture, increasing firmness and post-harvest life [33]. In addition, the relationship between phenol levels and the positive evaluation regarding color (Figure 1) can be attributed to the high correlation between these compounds and the existence of pigments with high antioxidant capacity, such as betacyanin, betaxanthin, and betalain [34,35]. Again, choices regarding the antioxidant capacity of the species used can be targeted, increasing the quality of the product sold to the final consumer.

## 4. Materials and Methods

In 2023, two experiments were carried out at the University of Talca’s Soilless Cultivation and Vertical Agriculture Unit, located at the Talca Campus in the Maule Region, South Central of Chile (35°24′26″ SL and 71°38′10″ WL). The experiments took place from April to May (autumn) and August to September (spring) and involved cultivating eleven different horticultural species as microgreens in rigid plastic trays (55 cm long × 28.5 cm wide) filled with moistened peat substrate up to 2 cm high (Table 7). These eleven species were selected because they are among the most common vegetables grown in the South-Central region of Chile. The rationale behind using a diverse range of species was to observe variations in the production process, product quality, and consumer acceptance, providing a comprehensive understanding of how different microgreens perform under controlled soilless cultivation conditions.

**Figure 4:**
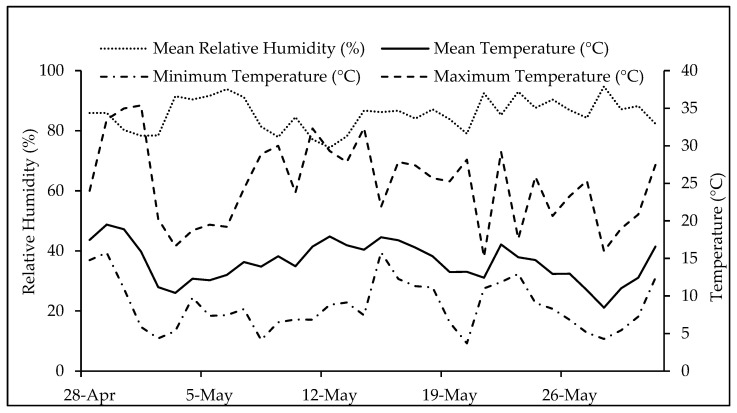
Relative humidity values and maximum, mean, and minimum temperatures during the autumn experiment.

**Figure 5:**
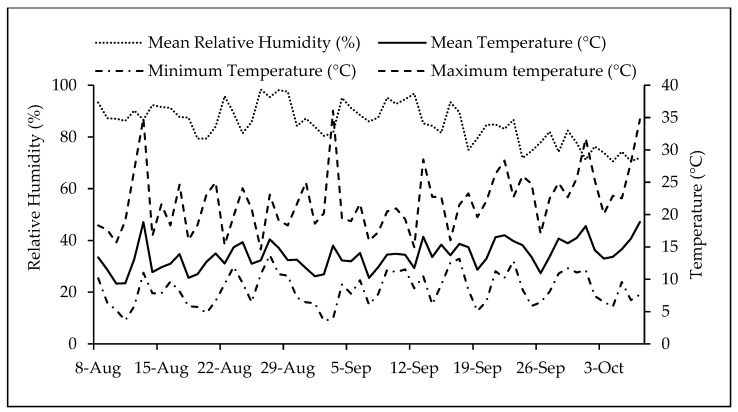
Relative humidity values and maximum, mean, and minimum temperatures during the spring experiment.

The seeds were sown manually in the trays and then covered until they sprouted. They received daily watering, and a nutrient solution with a low EC of 1.3 dS m^−1^ was applied every three waterings. Temperature and relative humidity in the greenhouse were measured using sensors; these variables ranged from 3.7 °C to 35 °C and 34% to 95% in the autumn experiment and 3.6 °C to 36.6 °C and 23% to 95% in the spring experiment, respectively (Figure 4 and Figure 5).

In each experiment, a completely randomized design with six replicates was implemented, with one tray assigned to each species as the experimental unit. After 33 days from sowing, the microgreens were harvested, and detailed plant height (cm) and fresh weight (g) measurements were taken as part of the agronomic evaluations. Following these measurements, the samples underwent chemical analysis to determine their nutritional content.

Total protein content (%) and nitrate levels (g L^−1^) were assessed using the Kjeldahl method, following the A.O.A.C. Official Methods of Analysis [36]. Total phenols (ppm of GAE) were quantified using the Folin–Ciocalteu method as outlined in the A.O.A.C. Official Methods of Analysis [36]. For β-carotene content (mg equiv 100 g^−1^), an organic solvent miscible in water, was employed to extract carotenoids, ensuring accurate quantification. Crude fiber content (%) was determined by the gravimetric method using a Velp fiber extractor [36,37,38].

Additionally, mineral contents—including iron (Fe), magnesium (Mg), calcium (Ca), sodium (Na), and phosphorus (P)—were measured in mg kg^−1^ using the electrothermal atomic absorption (EAA) method with Analytik Jena NOVAA 800 equipment. However, due to the small sample size in the spring experiment, only one repetition per species was conducted for chemical determination, which prevented statistical analysis of these variables.

To assess consumer acceptance, a sensory panel was conducted with 57 evaluators for the autumn experiment and 22 for the spring experiment. The panels evaluated all species based on flavor, texture, general appearance, and color using a nine-point hedonic scale, where one indicated “extremely pleasant” and nine indicated “extremely unpleasant” [39] (Table 8). In addition, consumers’ purchase intentions and habitual consumption patterns were evaluated using a five-point scale (Table 8).

**Table 8:** Code and definition of the sensory scale and purchase preference for microgreens.

Code	Sensory Scale	Purchase Preference Scale
1	Extremely pleasant	I would definitely buy it
2	Very pleasant	I would probably buy it
3	Moderately pleasant	May be maybe not
4	Sightly pleasant	I probably would not buy it
5	It does not matter to me	I definitely would not buy it
6	Slightly unpleasant	-
7	Moderately unpleasant	-
8	Very unpleasant	-
9	Extremely unpleasant	-

Statistical analyses of the parametric variables were carried out using analysis of variance (ANOVA), while non-parametric variables were subjected to the Kruskal–Wallis test. When significance was found, mean separation was performed using the Tukey Honest Significant Difference (HSD) test (*p* = 0.05). Additionally, a correlation network analysis was conducted using the Rbio program to explore relationships between variables [40].

## 5. Conclusions

This study demonstrates that cultivating various microgreen species in unheated greenhouses in South Central Chile is a viable strategy to enhance both nutritional quality and consumer acceptance of fresh produce. The findings confirm that microgreens produced under these conditions consistently exhibit high levels of essential nutrients and bioactive compounds, such as proteins, phenols, and beta-carotene, which are critical for promoting human health. Notably, the variation in nutritional composition among species, such as the high phenol and β-carotene content observed in Swiss chard, carrots, and species from the Brassicaceae family, underscores the importance of selecting specific cultivars to target desired nutritional and functional outcomes. These compounds are particularly valued for their antioxidant activity, with β-carotene also contributing anti-inflammatory effects and a high vitamin A content, making these microgreens a potent functional food. The agronomic evaluation revealed that radish had the highest yield among the evaluated species, further highlighting its potential for commercial production. Additionally, the sensory evaluation indicated a strong consumer preference for microgreens, demonstrating a strong relationship between β-carotene content and appearance, as well as magnesium content with taste and texture. This interaction between organoleptic perception and nutritional composition reinforces the appeal of microgreens as a health-promoting food choice. From a broader perspective, this research validates the potential of microgreens as a healthy, nutritious, productive, and sustainable food option, offering an alternative solution to global food security challenges. The high acceptance of these products among local consumers suggests a significant opportunity for their expansion in regional markets. However, future research should address challenges related to exploring additional species, optimizing post-harvest handling, and further elucidating their direct benefits to human health. Finally, the successful cultivation of microgreens in unheated greenhouses not only enhances food diversity and nutritional quality but also presents a sustainable agricultural practice that aligns with local market demands. Ongoing research and development should focus on refining cultivation practices, expanding species diversity, and enhancing post-harvest strategies to maximize microgreens’ health benefits and market potential, thereby contributing to more resilient and sustainable food systems.

## Figures and Tables

**Table 1:** Characteristics of germination rate, sowing density, and germination time of the species used to grow microgreens.

	Commercial Name	Sowing Density (g m^−2^)	Germination Index (%)	Germination Time (days)
Experiment 1	Chard	640	20	9
	Carrot	130	85	9
	Radish	320	85	3
	Lettuce	90	85	8
	Kale	190	85	3
	Arugula	128	95	3
Experiment 2	Mizuna	255	100	3
	Fennel	155	80	8
	Spinach	255	100	3
	Broccoli	320	95	3
	Anise	180	75	8

**Table 2:** Average height values harvested fresh mass and productivity of different species used to grow microgreens.

Commercial Name	Height (cm) *	Fresh Mass (g tray^−1^) *	Yield (g m^−2^) *
Autumn experiment			
Chard	3.6 d	116 bc	756 bc
Carrot	2.9 e	96 c	624 c
Radish	6.6 a	341 a	2216 a
Lettuce	2.9 e	295 a	1994 a
Kale	4.4 c	88 c	673 c
Arugula	6.2 b	136 b	1171 b
*p*-value	0.000	0.000	0.000
Spring experiment			
Mizuna	7.9 a	1367 a	8877 a
Fennel	8.4 a	808 b	5247 b
Spinach	6.7 b	995 b	6461 b
Broccoli	8.5 a	1346 a	8805 a
*p*-value	0.004	0.003	0.000

* The means followed by the same letter in the column do not differ from each other using the Tukey test at a 5% probability.

**Table 3:** Average values of total protein, total phenols, nitrates, raw fiber, and beta-carotene of different species used to grow microgreens.

Commercial Name	Total Proteins (%)	Total Phenols (mg kg^−1^ of GAE)	Nitrates (g L^−1^)	Raw Fiber (%)	β-Carotene (mg equiv 100 g^−1^)
Autumn experiment ^1^					
Chard	4.4 a	3335.9 a	3281.9 a	0.5 a	39,966 a
Carrot	4.6 a	3083.6 a	2508.5 a	0.5 a	15,379 d
Radish	3.7 a	2001.2 b	1099.5 b	0.6 a	17,438 c
Lettuce	3.8 a	2060.5 b	4169.3 a	0.7 a	12,550 e
Kale	4.6 a	3269.6 a	2489.1 a	0.6 a	19,369 b
Arugula	4.8 a	3304.6 a	2442.2 a	0.4 a	12,612 e
*p*-value	n.s.	0.007	0.000	n.s.	0.000
Spring experiment ^2^					
Mizuna	2.94	5663.8	4707.8	0.5	1451
Fennel	3.50	6164.5	5600.0	0.6	3474
Spinach	2.42	11,025.0	3866.7	0.5	3629
Broccoli	2.67	4538.9	4266.0	0.5	2498

GAE: Gallic acid equivalent; n.s.: not significant. ^1^ For the autumn experiment, means followed by the same letter in the column do not differ using the Tukey test at a 5% probability. ^2^ For the spring experiment, no statistical analyses were performed.

**Table 4:** Nutrient values of different species used for the production of microgreens.

Commercial Name	Fe (mg kg^−1^)	Mg (mg kg^−1^)	Ca (mg kg^−1^)	Na (mg kg^−1^)	P (mg kg^−1^)
Autumn experiment ^1^					
Chard	70.6 bc	207.4 bc	308.9 c	32.9 a	1.1 a
Carrot	75.8 bc	203.3 bc	313.6 c	26.3 ab	1.1 a
Radish	79.9 b	202.2 c	410.9 b	23.5 b	0.9 a
Lettuce	156.8 a	268.5 ab	353.1 c	26.2 ab	1.0 a
Kale	80.6 b	300.0 a	552.8 a	26.2 ab	1.2 a
Arugula	69.1 c	202.6 bc	380.7 bc	25.7 ab	0.8 a
*p*-value	0.000	0.001	0.000	0.015	n.s.
Spring experiment ^2^					
Mizuna	79.2	292.1	846.3	25.7	0.9
Fennel	54.5	279.4	587.6	25.9	0.4
Spinach	33.8	207.8	281.6	31.1	0.4
Broccoli	68.4	253.7	252.1	5.6	0.4

n.s.: not significant. ^1^ For the autumn experiment, means followed by the same letter in the column do not differ using the Tukey test at a 5% probability. ^2^ For the spring experiment, no statistical analyses were performed.

**Table 5:** Average values of sensory evaluations regarding appearance, color, texture, and flavor of different species used to produce microgreens.

Commercial Name	Appearance *	Color *	Texture *	Flavor *
Autumn experiment				
Chard	2.3 a	2.1 a	3.0 a	4.3 ab
Kale	2.7 ab	2.3 ab	2.8 a	3.2 a
Lettuce	2.9 ab	3.1 c	2.6 a	3.5 ab
Radish	3.0 ab	2.9 bc	3.2 a	4.5 b
Arugula	3.1 b	2.6 abc	3.2 a	4.5 b
Carrot	3.2 b	2.6 abc	3.2 a	3.7 ab
*p*-value	0.000	0.000	n.s.	0.000
Spring experiment				
Mizuna	2.2 a	2.0 b	2.6 ab	3.4 ab
Fennel	2.7 ab	2.7 ab	3.1 a	4.4 a
Spinach	3.2 b	3.3 a	2.2 b	2.7 b
*p*-value	0.05	0.02	0.05	0.01

n.s.: not significant. * The means followed by the same letter in the column do not differ from each other using the Tukey test at 5% probability. Each attribute was evaluated from 1 (extremely pleasant) to 9 (extremely unpleasant) (Table 8).

**Table 6:** Summary of consumers’ purchasing preferences regarding the different species used to produce microgreens.

Commercial Name	Purchase Intention *	Result of Purchase Intention
Autumn experiment		
Chard	2.2 a	I would probably buy it
Kale	2.4 a	I would probably buy it
Lettuce	2.5 a	I would probably buy it
Radish	2.6 a	I would probably buy it
Arugula	2.8 a	I would probably buy it
Carrot	2.8 a	I would probably buy it
*p*-value	n.s.	
Spring experiment		
Mizuna	2.0 a	I would definitely buy it
Fennel	2.6 a	I would probably buy it
Spinach	2.5 a	I would probably buy it
*p*-value	n.s.	

n.s.: not significant. * The means followed by the same letter in the column do not differ using the Tukey test at a 5% probability.

**Table 7:** General characteristics of the species used to produce microgreens.

	Commercial Name	Scientific Name	Commercial Variety
Autumn experiment	Chard	*Beta vulgaris* L. var*. cicla*	Penca roja
	Carrot	*Daucus carota* L.	Chantenay Royal 2
	Radish	*Raphanus sativus* L.	Pablo
	Lettuce	*Lactuca Sativa* L.	Tango roja
	Kale	*Brassica oleracea* L. var*. sabellica*	Verde rizado
	Arugula	*Eruca sativa* Mill.	Astro
Spring experiment	Mizuna	*Brassica rapa* L. subsp. *nipposinica*	Verde
	Fennel	*Foeniculum vulgare* L.	Etrusco
	Spinach	*Spinacia oleracea* L.	Woodduck
	Anise	*Pimpinella anisum* L.	-
	Broccoli	*Brassica oleracea* L. var*. italica*	Italian Green Sprouting

## Data Availability

This article was written within the framework of confidential projects, which mandate the non-disclosure of data generated and analyzed. Consequently, the data supporting the reported results are not publicly available due to privacy restrictions.
